# Expanding the spectrum of post-HSCT hemolysis: from passenger lymphocyte syndrome to host- and graft-associated alloimmune hemolytic anemia

**DOI:** 10.1007/s44313-026-00173-y

**Published:** 2026-09-16

**Authors:** Dong Wook Jekarl, Jungjun Lee, Yujin Cha, Jay Ho Han, Seung Hee Cho, Jihyang Lim, Yonggoo Kim

**Affiliations:** 1 Department of Laboratory Medicine, Seoul St. Mary’s Hospital, College of Medicine, The Catholic University of Korea, 222, Banpo-Daero, Seocho-Gu, Seoul, 06591 Republic of Korea; 2 College of Medicine, The Catholic University of Korea, Seoul, Republic of Korea; 3 Department of Laboratory Medicine, Uijeongbu St. Mary’s Hospital, College of Medicine, The Catholic University of Korea, Seoul, Republic of Korea; 4 Department of Laboratory Medicine, Eunpyeong St. Mary’s Hospital, College of Medicine, The Catholic University of Korea, Seoul, Republic of Korea; 5 Research and Development Institute for In Vitro Diagnostic Medical Devices, College of Medicine, The Catholic University of Korea, Seoul, Republic of Korea

**Keywords:** Hematopoietic stem cell transplantation, Hemolysis, Passenger lymphocyte syndrome, Alloimmunization

## Abstract

Immune-mediated alloimmunization and hemolytic anemia can complicate ABO-incompatible hematopoietic stem cell transplantation (HSCT). After major ABO-incompatible HSCT, clinical manifestations may include pure red cell aplasia and delayed hemolysis. Passenger lymphocyte syndrome and delayed hemolysis are typical complications of minor ABO-incompatible HSCT. Because delayed hemolysis after major or minor ABO mismatch may be indistinguishable, the terms host-associated alloimmune hemolytic anemia and graft-associated alloimmune hemolytic anemia have been tentatively proposed to distinguish these conditions according to their host or graft origin. However, the rarity of these subgroups may limit the clinical utility of this classification. Management across the clinical spectrum includes transfusion support, plasmapheresis, and therapeutics related to immunomodulatory therapies.

Hematopoietic stem cell transplantation (HSCT) involves the replacement of a recipient’s hematopoietic and immune systems with those of a donor and has been used to treat high-risk hematologic malignancies and non-malignant disorders for more than three decades [1–3].

The use of HSCT as a potentially curative therapy has increased over time. In Korea, 3,195 HSCTs were performed in 2024. Between 1983 and 2024, 52,112 transplantations were reported. The numbers of transplantations by graft source were as follows: autologous, 22,580; sibling, 12,171; unrelated, 10,301; haploidentical, 6,010; and cord blood, 1,050 [4]. The indications for HSCT during this period included (disease, number of cases [percentage]): acute myeloid leukemia, 12,941 (24.9%); multiple myeloma, 9,425 (18.1%); lymphoma, 8,965 (17.2%); acute lymphoblastic leukemia, 6,718 (12.9%); myelodysplastic syndrome, 3,723 (7.0%); aplastic anemia, 3,092 (6.1%); chronic myeloid leukemia, 1,188 (2.4%) and other diagnoses, 6,057 (11.9%) [4].

Prior to the infusion of autologous or allogeneic hematopoietic stem cells, the recipient’s bone marrow and immune compartments are suppressed or eradicated using conditioning regimens. Myeloablative conditioning involves high-dose chemotherapy with agents such as busulfan, cyclophosphamide, or the use of total-body irradiation to eradicate the host’s hematopoietic system.

Reduced-intensity conditioning (RIC) uses lower doses or less toxic combinations of fludarabine with busulfan or melphalan to achieve sufficient immunosuppression for engraftment without complete marrow ablation [5–7].

Despite its therapeutic benefits, HSCT is associated with significant toxicities, including infection, organ failure, graft-versus-host disease (GVHD), and hemolysis. In contrast to solid organ transplantation, in which the primary immunologic concern is host rejection of the graft, HSCT management focuses on limiting donor immune-mediated injury to the recipient (GVHD) [8, 9]. Acute GVHD is principally mediated by donor T lymphocytes that recognize host antigen-presenting cells and commonly involves the skin, gastrointestinal tract, and liver. Chronic GVHD typically emerges approximately 100 days after HSCT, manifests with features resembling autoimmune and sclerotic diseases and may cause fibrosis in the affected organs. Frequently involved organs and tissues include the skin, oral mucosa, liver, eyes, and lungs [8–11].

Matching at the HLA-A, -B, -C, and -DRB1 loci is critical for the success of related and unrelated donor HSCT, whereas HLA mismatch is associated with an unfavorable outcomes [12, 13]. Donor selection depends primarily on HLA compatibility and graft source [14]. The preferred hierarchy is as follows: HLA-identical sibling donors, fully matched unrelated donors, mismatched familial donors (haploidentical donors), and mismatched unrelated donors. The frequency of haploidentical HSCT has increased with efforts to expand the potential donor pool [4]. The use of granulocyte colony-stimulating factor-mobilized grafts combined with antithymocyte regimens or post-transplantation cyclophosphamide has broadened the applicability of haploidentical HSCT. High-resolution HLA typing using massively parallel sequencing (next-generation sequencing) has improved the accuracy of HLA assignments in HSCT [15, 16].

In contrast to HLA disparity, outcomes after ABO-incompatible HSCT show inconsistent effects on overall survival and GVHD risk [17, 18]. However, ABO incompatibility can lead to specific complications, most notably immune hemolysis.

## Major ABO-incompatible HSCT

**Fig. 1:**
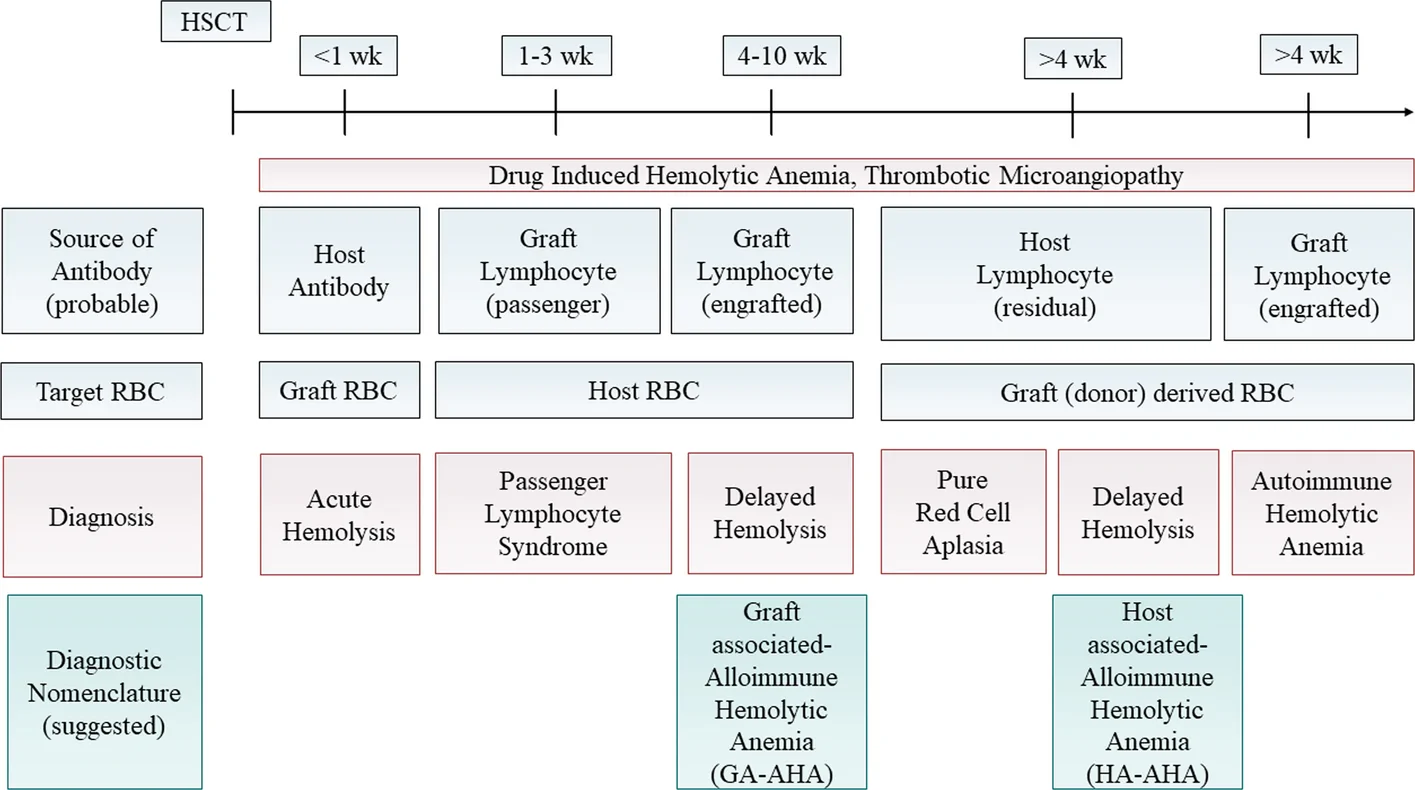
Hemolytic anemia and pure red cell aplasia after HSCT. Adverse events possibly occurring noted on a temporal sequence. Nomenclatures are suggested for delayed hemolysis of graft (donor) derived RBC [23–25] and delayed hemolysis of host (recipient) RBC [26–30]. Abbreviation: HSCT, hematopoietic stem cell transplantation; RBC, red blood cell; wk, week

Major ABO-incompatibility is defined as the presence of host (recipient) isoagglutinins directed against ABO antigens on donor cells. Examples include donor and recipient (donor ABO recipient ABO) combinations such as A→, B→,→ O, AB,→ A, AB,→ B, A, B, B→ A [19, 20]. ABO-incompatible HSCT accounts for 30% of related donors and up to 50% of unrelated donors [20–22] and presents several post-transplant challenges, including immune-mediated hemolysis (Fig. 1).

### Acute hemolysis

In major ABO-incompatible HSCT, host (recipient) isoagglutinins can mediate acute hemolysis of graft (donor) red blood cells (RBCs) present in the stem cell graft [31–33]. Preventive measures include reducing the number of RBC in the graft by centrifugation. Pretransplant plasmapheresis or immunoadsorption can be performed to lower host isoagglutinin titers. Supportive measures, such as transfusion of compatible RBCs may be required until erythropoiesis is reconstituted and hemoglobin levels stabilize [19, 33].

### Pure red cell aplasia

Host isoagglutinin directed against donor RBC antigens can suppress erythroid recovery after HSCT, resulting in marked reticulocytopenia without overt hemolysis. The reported incidence ranges from 0% to 29% [33–35]. In one pooled series, 11.7% (83/707) of patients developed pure red cell aplasia (PRCA), and 15.7% (13/83) had concomitant pancytopenia [33–35]. The use of fludarabine with busulfan as the conditioning regimen was identified as a risk factor, with an odds ratio (95% confidence interval) of 8.18 (2.07–31.14). These findings have been attributed to the persistence of host plasma cells [33–35]. Isoagglutinin levels declined more slowly in affected patients than in controls. Erythroid engraftment was selectively delayed, whereas myelopoiesis and thrombopoiesis showed normal recovery kinetics [33–36].

### Major blood group mismatch

A major blood group mismatch can be defined as the presence of a blood group in the graft (e.g., Rhesus E [RhE]-positive) that is absent in the host (e.g., RhE-negative). Major blood group mismatches include all major ABO incompatibilities (host isoagglutinins directed against graft antigens) and mismatches involving other blood group antigens that are present in the graft but absent in the host.

### Delayed hemolysis or host-associated alloimmune hemolytic anemia

Delayed hemolysis after major ABO-mismatch HSCT is mediated by long-lived host plasma cells that produce alloantibodies directed against engrafted RBCs despite conditioning. Unlike PRCA, this subgroup manifests with overt hemolysis (Table 1). Hemolysis, characterized by a positive direct antiglobulin test and abrupt decreases in hemoglobin and haptoglobin levels, may occur [26–30].

**Table 1:** Case reports of delayed hemolytic anemia due to host (recipient) remnant lymphocyte (Host-associated alloimmune hemolytic anemia, HA-AHA)

Serial	Year	Age	Sex	Diagnosis	Transfusion	Donor	Recipient	Author
1	1998	41	M	CML	NA	A Jkb( +)	O Jkb(-)	Lopez A [26]
2	2003	26	M	AA	NA	O ccDEE	B CCDee	Izumi N [27]
3	2007	6	M	ALD	NA	A	O-	Chao MM [28]
4	2015	57	M	MDS	NA	A	O	Hosoba S [29]
5	2018	62	F	MDS	NA	O CcEe	AB Ccee	Isobe M [30]

*CML* Chronic myeloid leukemia, *AA* Aplastic anemia, *ALD* adrenoleukodystrophy, *HA-AHA* host-associated alloimmune hemolytic anemia, *MDS* myelodysplastic syndrome

 Alloantibodies targeting donor-derived RBCs are detected as increased antibody titers or in eluate. The reported specificities of host-derived alloantibodies include anti-A, anti-JKb [26], anti-E, anti-c [27], anti-A [28, 29], and anti-E [30] (Table 1).

In contrast, post-HSCT autoimmune hemolytic anemia (AIHA) is driven by engrafted (donor-derived) lymphocytes and antibodies directed against engrafted RBCs. Drug-induced hemolysis and thrombotic microangiopathy should also be excluded. Because delayed hemolysis after major or minor ABO mismatch can be clinically indistinguishable, the term “host-associated alloimmune hemolytic anemia” (HA-AHA) was tentatively proposed to denote host (recipient)-mediated alloimmune hemolysis. In the absence of hemolysis, host-associated alloimmunization may be used to describe this subgroup. However, owing to the rarity of this subgroup, its practical utility is limited. It is possible that HA-AHA may have been underdiagnosed or misdiagnosed.

In HA-AHA, alloantibodies can be produced through the immunization of residual host lymphocytes. HA-AHA occurs because of a major blood group mismatch. If an alloantibody (e.g., anti-E) is detected, antibodies from intravenous immunoglobulin (IVIGV), Rhesus D (RhD) immunoglobulin, or other sources should be ruled out. After confirming bone marrow engraftment by biopsy and considering the timeline (> 4 weeks, usually 4–6 month after HSCT), the source of alloantibodies (anti-E) is expected to be the host. Hemolysis without autoreactivity can be regarded as HA-AHA. The production of these alloantibodies is inferred from their detection in the host prior to HSCT [27–30, 37] or shortly after HSCT [26], which is consistent with a host origin.

The pathophysiology of HA-AHA is unclear; however, it may parallel antibody-mediated rejection as described in solid organ transplantation. Unlike antibody-mediated rejection, which targets donor parenchymal and endothelial cells within the graft, HA-AHA involves host-derived alloantibodies directed against RBC antigens of donor origin.

## Minor ABO incompatible HSCT

Minor ABO-incompatibility is defined as the presence of graft(donor) isoagglutinin directed against the host (recipient) ABO antigen. Typical donor and recipient combination (donor ABO, recipient ABO) is as follows: O, A; O, B; O, AB; A, AB; B, AB; A, B; B, A, respectively [19–21].

### Minor blood group mismatch

A minor blood group mismatch can be defined as a graft without the expression of certain blood group antigens (e.g., RhE-negative), while the corresponding blood group antigen is expressed by the host (e.g., RhE-positive). Minor blood group mismatches include all minor ABO incompatibilities (isoagglutinins against the host), except for bidirectional incompatibility and other blood group antigens expressed by the host but absent in the graft.

### Passenger lymphocyte syndrome (PLS)

PLS occurs in the context of minor blood mismatches. The antibodies in PLS derived from the graft (the product collected from the donor), which does not express certain blood group antigens (e.g., blood group O), whereas the recipient expresses them (for example, blood group A). If anti-A is detected, anti-A from intravenous immunoglobulin (IVIG), RhD immunoglobulin, or other sources should be excluded. After confirming that the timeline is too early for engraftment (PLS usually occurs approximately 1–2 weeks after HSCT) and confirming the presence of hemolysis, PLS can be suspected. The interaction of graft-derived antibodies (donor lymphocytes) with host (recipient) RBCs can cause complement-mediated hemolysis and occasionally cytopenia [36].

The reported specificities of the antibodies include anti-A, anti-B, anti-RhD, and anti-Kidd (anti-JK) antibodies [19–21]. Risk factors for PLS include peripheral blood stem cell grafts (vs. bone marrow), cyclosporine-only GVHD prophylaxis, an RIC regimen, and blood group O donors with blood group A recipients [19–21]. Hemolysis typically presents approximately 1–3 weeks after HSCT.

The diagnosis of PLS is based on an acute decline in hemoglobin levels with evidence of immune-mediated hemolysis. Diagnosis is based on the detection of host (recipient) RBC-targeted isoagglutinins or antibodies, a positive direct antiglobulin test, the presence of antibodies in the eluate, elevated lactate dehydrogenase levels, and decreased haptoglobin levels. Management may include transfusion of blood group O RBCs, with or without washing; therapeutic plasmapheresis or immunoadsorption to reduce circulating antibodies; RBC exchange with the donor type; and immunotherapies such as rituximab, corticosteroids, and IVIG [19–21].

For the collection of case series, PubMed, Web of Science, Google Scholar, and Embase were searched using the terms “PLS,” “hemolysis,” and “HSCT.” Reports related to solid organ transplantation and non-English-language publications were excluded. The pooled case series is summarized in Table 2 [37–67].

**Table 2:** Case reports of PLS. RhD negative blood groups are noted as a bar. Transfusion indicates units of RBC transfused for PLS treatment

Serial	Year	Age	Sex	Diagnosis	Transfusion	Donor	Recipient	Author
1	1986	5	M	AML	11	O	A	Haas RJ [37]
2	1986	17	M	CML	9	O	A-	Hows J [38]
3	1986	13	M	CML	6	O	A	Hows J
4	1986	24	M	SAA	14	O	B	Hows J
5	1986	11	F	AML	7	O	A	Hows J
6	1986	26	M	ALL	6	B	AB	Hows J
7	1986	32	F	SAA	5	**A-**	A	Hows J
8	1992	35	NA	CML	26	O	A	Gajewski JL [39]
9	1992	45	NA	CML	26	O	A	Gajewski JL
10	1992	47	NA	CML	26	A	B	Gajewski JL
11	1992	21	NA	ALL	16	A	B	Gajewski JL
12	1992	22	NA	AML	10	O	B	Gajewski JL
13	1992	35	NA	AML	10	O	A	Gajewski JL
14	1992	25	NA	CML	6	O	A	Gajewski JL
15	1996	12	NA	ALL	7	O	A	Toren A [40]
16	1996	37	F	CML, BC	16	O	A	Greeno EW [41]
17	1997	23	F	CML, AP	10	B	A	Bornhauser M [42]
18	1997	38	M	MM	12	O	A	Oziel-Taieb S [43]
19	1997	19	M	AML	NA	NA	NA	Moog R [44]
20	1997	37	M	MM	9	O	A	Laurencet FM [45]
21	1998	50	M	AML	NA	A	B	Franchini [46]
22	1999	16	M	AML	7	O	A	Salmon JP [47]
23	2000	50	F	AML	10	A	A	Leo A [48]
24	2001	28	M	ALL	17	O	A	Tiplady CW [49]
25	2001	37	M	CML	16	A	B-	Young PP [50]
26	2001	39	F	NHL	10	O	O	Young PP
27	2001	55	M	CLL	9	O	A	Bolan CD [51]
28	2001	38	F	AML	8	O	B	Bolan CD
29	2001	38	M	NHL	4	O	A	Bolan CD
30	2002	7	M	ALL	7	O	A	Hoegler W [52]
31	2002	35	M	ALL	NA	O	A	Worel N [53]
32	2002	33	M	AML	NA	O	A	Worel N
33	2002	47	F	NHL	NA	O	B	Worel N
34	2002	41	M	AML	NA	O	A	Worel N
35	2003	35	M	CTCL	2	O	B	Noborio K [54]
36	2003	61	M	AML	NA	O	A	Reed M [55]
37	2005	54	M	TPLL	8	O	A	Curtin NJ [56]
38	2005	38	M	CML	10	B	A	Benson DM [57]
39	2007	13	F	ALL	31	O	A	Nair V [58]
40	2008	65	F	CMMoL	7	O	A	Lee HJ [59]
41	2010	58	M	CML, BC	12	A-	O	Adam BR [60]
42	2012	16	M	AML	2	O	A	Iwanaga S [61]
43	2013	59	F	DLBCL	NA	O	A	Zaimoku Y [62]
44	2013	58	M	MDS	NA	O	A	Zaimoku Y
45	2013	48	F	PTLC	NA	A	B	Zaimoku Y
46	2013	56	F	CML	NA	O	A	Zaimoku Y
47	2013	56	F	AML	NA	A	B	Zaimoku Y
48	2013	53	M	MDS	2	O	A	Akkok CA [63]
49	2014	58	M	CML, BC	12	A-	O	Squires JE [64]
50	2018	14	M	MPS	1	O	A	Xu Z [65]
51	2021	38	F	MPAL	4	B	O	Teshigawara-Tanabe H [66]
52	2021	38	F	MPAL	7	A	O	Teshigawara-Tanabe H
53	2021	20	M	HL	4	A	B	Teshigawara-Tanabe H
54	2022	24	M	ALL	7	O	A	Noubouossie DF [67]

*HSCT* hematopoietic stem cell transplantation, *AML* acute myeloid leukemia, *CML* chronic myeloid leukemia, *SAA* severe aplastic anemia, *ALL* acute lymphoblastic leukemia, *BC* blast crisis, *ap* acute phase, *MM* multiple myeloma, *NHL* non-Hodgkin’s lymphoma, *CLL* chronic lymphocytic leukemia, *CTCL* cutaneous T cell lymphoma, *CMMoL* chronic myelomonocytic leukemia, *DLBCL* diffuse large B cell lymphoma, *MDS* myelodysplastic syndrome, *PLS* passenger lymphocyte syndrome, *PTLC* peripheral T cell lymphoma, *RhD* Rhesus D, *MPS* mucopolysaccharidoses, *MPAL* mixed phenotype acute leukemia, *HL* Hodgkin’s lymphoma

### Delayed hemolysis or graft-associated alloimmune hemolytic anemia

In contrast to PLS, delayed immune-mediated hemolysis typically occurs approximately 4 weeks after HSC engraftment. Antibodies are produced by engrafted donor-derived lymphocytes and are directed against host (recipient) RBC blood group antigens [23–25]. Because the clinical presentation may be indistinguishable from delayed hemolysis following major ABO mismatch, the term “graft-associated alloimmune hemolytic anemia” (GA-AHA) is tentatively proposed. If hemolysis is absent, the term graft-associated alloimmunization might be used. However, given the rarity of this subgroup, the practical utility of this nomenclature may be limited (Table 3).

**Table 3:** Case reports of delayed hemolytic anemia due to engrafted donor lymphocyte (Graft-associated alloimmune hemolytic anemia, GA-AHA)

Serial	Year	Age	Sex	Diagnosis	Transfusion	Donor	Recipient	Author
1	2004	56	M	RCC	10	A-	A	Contentin N [23]
2	2017	31	F	HL	11	AB-	A	Bailen R [24]
3	2021	17	M	MM	9	A2-	A1-	Hecker JS [25]

*GA-AHA* graft associated alloimmune hemolytic anemia, *RCC* renal cell carcinoma, *HL* Hodgkin’s lymphoma, *MM* multiple myeloma

The presence of hemolysis should be noted, and a differential diagnosis including PLS, drug-induced hemolysis, and thrombotic microangiopathy should be performed. The diagnosis is based on the detection of antibodies targeting host (recipient) RBCs, alloantibodies, increased isoagglutinins levels, and the results of the direct Coombs test. In the reported cases, anti-D [23], anti-D and anti-A1 [24], and anti-A1 [25] antibodies produced by engrafted lymphocytes were detected.

The pathophysiology of GA-AHA remains poorly defined; however, it may represent a hematological manifestation within the spectrum of GVHD after HSCT. While classical acute GVHD predominantly affects the skin, gastrointestinal tract, liver, and other parenchymal tissues, GA-AHA involves alloantibodies produced by engrafted donor-derived lymphocytes that target host RBC. Considering that approximately 4 weeks have elapsed after HSCT, it is reasonable to conclude that the antibodies originated from the engrafted bone marrow. Hemolysis can be regarded as GA-AHA. However, the antibodies may instead be produced by lymphocytes present in the donor graft, in which case the condition would be classified as PLS.

### Autoimmune hemolytic anemia

Post HSCT AIHA occurs in approximately 4%–6% of patients and may present with concurrent thrombocytopenia and/or neutropenia. Median onset ranges from 4 to 10 months after HSCT [21, 68–70]. Its pathogenesis is attributed to impaired T cell immunity due to immunosuppression, imbalanced B and T cell lymphopoiesis, and delayed T cell reconstitution [71]. Warm pan-reactive autoantibodies are the most common, followed by anti-Rh-specific autoantibodies [70]. Risk factors include unrelated donor graft, concurrent chronic GVHD, lymphocyte-depleted graft, in vivo lymphodepletion (by anti-thymocyte globulin or alemtuzumab), non-malignant underlying disease indicated for HSCT, omission of total body irradiation, cord blood graft, younger host age, and a short interval from diagnosis to HSCT [68–71].

### Differential diagnosis

We might attempt to classify a continuous spectrum of disease into discrete categories, although the boundaries between these categories are not always clear. However, the value of theis classification is that it enhances our understanding of diseases that occur after HSCT (Table 4).

**Table 4:** Differential diagnosis of hemolysis including pure red cell aplasia (PRCA) after hematopoietic stem cell transplantation

	Graft Ab	PLS	GA-AHA	PRCA	HA-AHA	AIHA
Estimated Occurrence (week)	< 1w	1-3w	4w-10w	< 1w	> 4w	> 4w
Major blood group mismatch	O	X	X	O	O	
Minor blood group mismatch	X	O	O	X	X	
Host RBC	O	O	O	O	X	X
Graft RBC	X	X	X	X	O	O
Engraftment	X	X	O	X	O	O
Hemolysis	O	O	O	X	O	O

*Ab* antibody, *RBC* red blood cell, *PLS* passenger lymphocyte syndrome, *GA-AHA* graft-associated alloimmune hemolytic anemia, *PRCA* pure red cell aplasia, *HA-AHA* host-associated alloimmune hemolytic anemia, *AIHA* autoimmune hemolytic anemia, *w* week

## Conclusion

ABO-incompatible HSCT is common because HLA compatibility is prioritized during donor selection. Host- or graft-driven alloimmunization and hemolysis may occur, resulting in various clinical manifestations. Clinical entities, including PLS, GA-AHA, PRCA, and HA-AHA, likely represent a continuum of alloimmunity with overlapping pathophysiologies. Evaluation of post-HSCT hemolysis requires a comprehensive approach to exclude alternative etiologies, such as drug-induced hemolytic anemia and thrombotic microangiopathy. In addition to isoagglutinins, antibodies directed against other RBC blood group antigens should also be considered potential causes of hemolysis. Prompt detection and diagnosis, followed by supportive management, including transfusion, plasmapheresis, immunoadsorption, and immunomodulatory therapies, are essential to mitigate clinical symptoms and improve outcomes.

## Data Availability

No datasets were generated or analysed during the current study.
